# Treatment of Hæmorrhoids

**Published:** 1871-04

**Authors:** 


### TREATMENT OF HÆMORRHOIDS.

Dr. Maclean read a paper on this subject before the British Medical Association. He remarked that Hæmorrhoids are extremely common, but that we are rarely called upon for advice until the disease has assumed a stage in which ordinary remedies are of no use, and active procedure is necessary. He referred to the description of Hæmorrhoids given by Erchisen, to the correctness of which he testified. From this it appears that all kinds of piles are composed of sacs or cells, with fluid contents; and so long as this remains, the suffering persists.—An obvious, though hitherto unnoticed, plan of treatment, consists in supporting the weakened walls and then emptying the sac in the mode usually adopted in the case of hernial tumors. He was led to the discovery of its efficacy from employing it in a case of piles occurring during labor. The method he proposes consists, first, in getting a free evacuation of the bowels by some aperient medicine, and when the effects of the medicine have passed off, he orders the parts to be well fomented for a few hours, to relieve as much as possible the irritation and spasms of the parts. He then proceeds to apply the taxis to the tumor. Taking a piece of soft, well-oiled cloth, and grasping one of the tumors—if there be more than one—with two fingers and the thumb, thereby encircling the enlargement, and curving the finger so that they cover the fundus of the pile, he proceeds to press the tumor towards the mouth of the sac, with a kneading motion, continuing for a little time, until he finds the swelling becomes gradually smaller under the manipulation, and there only remains the thickened integument and whatever effusion of serum may have taken place into the cellular tissue. In the beginning of the application of this process the pain is sometimes considerable; but as the tumor becomes emptied the pain decreases, and when fully reduced, a great sensation of relief is experienced. Having completed the reduction of the first hæmorrhoids, the same procedure is applied to the others in rotation; and the whole being reduced, astringent lotions or ointments are applied to the parts and the operation is complete.—*London Lancet.*

NEW VOLUME.—With this number begins a new volume. Those indebted to us for subscription will find a reminder, in the shape of an envelope, in the present number of our magazine. Please return it to us with subscription inclosed.

